# Biomimetic matrices for rapidly forming mineralized bone tissue based on stem cell-mediated osteogenesis

**DOI:** 10.1038/s41598-018-32794-4

**Published:** 2018-09-26

**Authors:** Marta S. Carvalho, Atharva A. Poundarik, Joaquim M. S. Cabral, Cláudia L. da Silva, Deepak Vashishth

**Affiliations:** 1 Center for Biotechnology and Interdisciplinary Studies, Department of Biomedical Engineering, Rensselaer Polytechnic Institute, Troy, NY USA; 2 Department of Bioengineering and iBB – Institute of Bioengineering and Biosciences, Instituto Superior Técnico, Universidade de Lisboa, Lisboa, Portugal; 3 The Discoveries Centre for Regenerative and Precision Medicine, Lisbon Campus, Instituto Superior Técnico, Universidade de Lisboa, Lisboa, Portugal

## Abstract

Bone regeneration, following fracture, relies on autologous and allogenic bone grafts. However, majority of fracture population consists of older individuals with poor quality bone associated with loss and/or modification of matrix proteins critical for bone formation and mineralization. Allografts suffer from same limitations and carry the risk of delayed healing, infection, immune rejection and eventual fracture. In this work, we apply a synergistic biomimetic strategy to develop matrices that rapidly form bone tissue - a critical aspect of fracture healing of weight bearing bones. Collagen matrices, enhanced with two selected key matrix proteins, osteocalcin (OC) and/or osteopontin (OPN), increased the rate and quantity of synthesized bone matrix by increasing mesenchymal stem/stromal cell (MSC) proliferation, accelerating osteogenic differentiation, enhancing angiogenesis and showing a sustained bone formation response from MSC obtained from a variety of human tissue sources (marrow, fat and umbilical cord). *In vivo* assessment of OC/OPN mineralized scaffolds in a critical sized-defect rabbit long-bone model did not reveal any foreign body reaction while bone tissue was being formed. We demonstrate a new biomimetic strategy to rapidly form mineralized bone tissue and secure a sustained bone formation response by MSC from multiple sources, thus facilitating faster patient recovery and treatment of non-union fractures in aging and diseased population. Acellular biomimetic matrices elicit bone regeneration response from MSC, obtained from multiple tissue sources, and can be used in variety of scaffolds and made widely available.

## Introduction

New promising solutions for bone reconstruction have been developed due to the increased clinical demand for tissue engineered bone^1^. In fact, each year in United States alone, more than one million non-union fractures are treated^1,2^. To date most common procedures for bone regeneration still rely on bone grafts, both autologous or allogeneic bone grafts^1^. However, these approaches have drawbacks and are not ideal for bone regeneration. In the case of autografts, possible complications may occur, such as pain, infection, scarring and patients will eventually experience fractures^1,3^. Allografts have also similar limitations, namely the higher risk of immunologic rejection, besides infection^3^.

Although bone has a regenerative capacity of healing without forming a fibrous scar, this biological process can fail, leading to delayed healing or development of non-union fractures, significantly impacting the economics and patient’s quality of life^4^. Acceleration of the fracture healing process would bring some benefits, such as the reduction of medical costs and enhancement of quality of life by decreasing pain and increasing patient’s mobility^4^. Even though materials science technology has resulted in clear improvements and breakthroughs for bone tissue engineering applications, challenges to achieve functional and mechanically competent bone growth remain^5^. In particular, it lacks a carefully crafted strategy, similar to one employed *in vivo*, to address various aspects of forming functional load bearing bone.

Bone formation *in vivo* is the result of different sequential stages that include the recruitment, migration and proliferation of osteoprogenitors cells from surrounding tissues followed by their osteoblastic differentiation, matrix formation and tissue mineralization^6^. It is known that most of the outstanding properties of the bone are related to its matrix constitution^7^. By looking deep into nature, we observe that most of the tissues are composed of collagen^8^. However, only few of these tissues like bone, containing distinct extracellular matrix (ECM) compositions, are mineralized. Therefore, the composition of the bone extracellular matrix defines its unique properties and bone matrix composition is indeed different from the others extracellular matrices in the organism.

Bone extracellular matrix has two components: a mineral part comprising hydroxyapatite (70–90%) and an organic part (10–30%) of primarily collagen (approx. 90% of organic matrix) with the rest being non-collagenous proteins (~10%)^7,9^. Collagen plays a critical role in the structure and function of bone tissue^9^. Within the group of non-collagenous proteins, osteocalcin (OC) and osteopontin (OPN) are the most abundant, representing 10–20% of the non-collagenous proteins^7^. Together, collagen and the non-collagenous matrix proteins allow for the deposition of hydroxyapatite.

During childhood and adolescence, bone growth process is most active and enables long bones to increase in diameter and to change shape. In adult vertebrates, bones are constantly being remodeled, due to the regulation of bone resorption and formation processes. Interestingly, when investigating protein contents in osteonal *versus* interstitial bone tissue, our group demonstrated that, compared to older bone, OC and OPN are found in higher levels in younger bone, highlighting the potential role and/or regulation of these non-collagenous proteins in bone formation, remodeling and mineralization^7^. Although it is well known that the skeletal tissue is controlled by hormonal regulation, bone non-collagenous proteins trapped within bone ECM have been reported to play a critical role in regulating the normal and pathological skeletal growth and remodeling^7,10^.

OPN is an arginine-glycine-aspartate (RGD)-containing adhesive glycoprotein^11^. This protein was first identified in bone matrix, however it can be detected in other tissues, such as dentin, cartilage, kidney, and vascular tissues^11^. OPN can bind to α_ν_β_3_ integrins through their RGD domain. Additionally, OPN can also present an RGD-independent mechanism, in which OPN may engage CD44^12^, a cell surface adhesion molecule, involved in cell-cell and cell-matrix interactions. OPN has been proposed to regulate many physiological processes such as collagen organization, cell adhesion, cell viability, cell migration, angiogenesis and calcification^7,11,13^. OC is the most abundant bone specific non-collagenous protein in bone extracellular matrix that has been conserved in bone through evolution. It has affinity for calcium, playing an important role in matrix mineralization^14^. OC also functions in cell signalling and the recruitment of osteoclasts and osteoblasts, having important roles in bone resorption and deposition, respectively^15^.

Recent studies conducted by our group and others have demonstrated the role of OPN and/or OC as structural molecules in bone matrix linking the organic and inorganic matrices and contributing to structural integrity of bone^7,16–18^. Moreover, loss and modification of OC and/or OPN from bone matrix, known to occur with tissue age^7^ and with aging in humans^19–22^, leads to loss of structural integrity^23^ and altered mineralization^13,24^. Thus, autografts as well as allografts, typically obtained from older donors or patients that were subjected to total hip arthroplasty procedures^25^, are likely to contain bone tissue that is structurally compromised and not fully functional to promote mineralization.

Bone marrow mesenchymal stem/stromal cells (BM MSC) have been suggested for cell-based tissue engineering therapies, due to their potent immunomodulatory properties, capacity for self-renewal and ability to differentiate into different cell lineages, such as bone, cartilage and fat^26,27^. Under defined conditions *in vitro* (e.g., dexamethasone, ascorbic acid, β-glycerophosphate), it is possible to direct MSC along an osteogenic lineage. During this phase, alterations in cellular morphology, proliferation and gene expression lead to secretion of an organized extracellular matrix on which calcium phosphate is deposited as hydroxyapatite crystals^28^.

**Figure 1:**
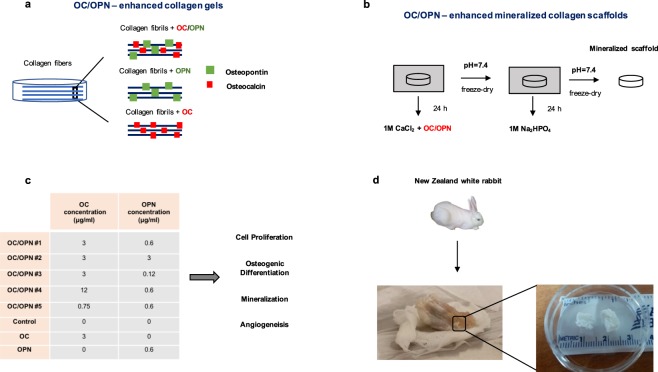
Design of the biomimetic OC/OPN-enhanced collagen matrices. (**a**) Schematic of OC/OPN-enhanced collagen gels. (**b**) Schematic of fabrication of OC/OPN-enhanced mineralized collagen scaffolds. (**c**) Different concentrations of OC and OPN incorporated into type I collagen gels at 3 mg/ml to create biomimetic matrices, based on the average of OC recovered from human bone^29^. Cell proliferation, osteogenic differentiation, mineralization and angiogenesis were the different variables evaluated after cell culture on OC/OPN-enhanced collagen gels. (**d**) Left: Representation of the OC/OPN-enhanced mineralized collagen scaffold implanted into a rabbit tibia after 6 weeks of surgery. Right: OC/OPN-enhanced mineralized collagen scaffolds prior to implantation into a rabbit model.

We posited that non-collagenous proteins from bone ECM, specifically OC and OPN, may be combined to design a novel biomimetic collagen matrix that accelerates bone healing response. To this end, we assess the synergistic effect of OC/OPN on different aspects of bone regeneration including MSC proliferation, osteogenic differentiation, mineralization and angiogenic properties (Fig. 1). We evaluated 5 different concentrations of OC/OPN combinations, above and below bone matrix physiological levels, and identified biomimetic OC/OPN-enhanced collagen matrices that enhance early osteogenic differentiation of MSC and sustain bone formation response (Fig. 1). The presence of these proteins on the matrix was confirmed by a SDS-PAGE protein gel and by measuring the amount of OC and OPN released from the collagen gels after 24 h and 21 days (Supplementary Figs 2 and 3). Data included in Supplementary Fig. 3 shows that OC and OPN are not being released from the gels after 21 days.

Effects of OC/OPN-enhanced collagen matrices were also evaluated on human MSC from different sources to address the broad application of this approach for bone regeneration. This study aims to develop a new strategy for rapidly forming and sustaining functional bone formation by utilizing OC and OPN and determining the mechanism for their synergistic effect on bone regeneration. To confirm the synergistic effect of these two bone ECM proteins, OC-enhanced collagen matrices and OPN-enhanced collagen matrices were also investigated.

## Results

### Effects of OC/OPN-enhanced collagen gels on MSC adhesion and proliferation

**Figure 2:**
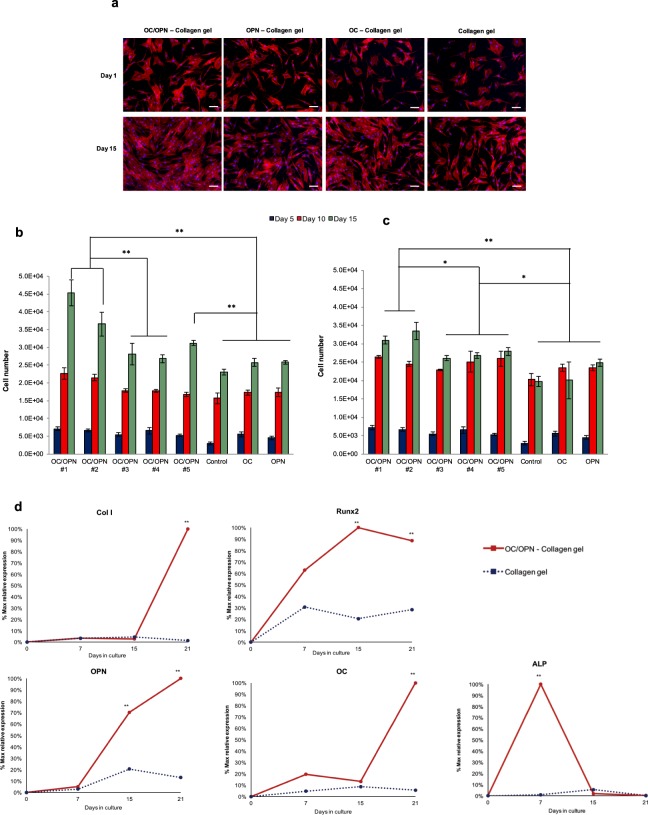
Effects of OC/OPN-enhanced collagen gels on cell proliferation and osteogenic differentiation of BM MSC. **(a**) Fluorescence microscopy images of DAPI (blue) and Phalloidin (red) of BM MSC seeded on top of different substrates 24 h and 15 days after cell seeding (OC/OPN #1) under DMEM + 10% FBS. Scale bar, 100 µm. (**b,c**) Proliferation of BM MSC cultured on OC/OPN-enhanced collagen gels for 15 days under (**b**) DMEM + 10% FBS and (**c**) osteogenic differentiation medium. (**d**) Percentage of maximum gene expression (collagen I [ColI], runx2, osteopontin [OPN], osteocalcin [OC] and alkaline phosphatase [ALP]) by BM MSC upon culture for 7, 15 and 21 days on control collagen gels and OC/OPN-enhanced collagen gels. Data are expressed as mean ± s.e.m.; **P < 0.01; *P < 0.05.

Fluorescence microscopy images of DAPI and phalloidin of BM MSC seeded on top of OC/OPN-enhanced collagen gels 24 hours after seeding suggested that the cells attached efficiently onto all substrates. Qualitatively, the cells were randomly distributed and displayed their native spread morphology (Fig. 2a). The capacity of the OC/OPN-enhanced collagen gels to promote BM MSC proliferation is shown in Fig. 2b,c for cells cultured using DMEM + 10% FBS (growth medium) and osteogenic medium (differentiation medium). After 15 days, BM MSC were present in higher number when cultured onto OC/OPN-enhanced collagen gels than onto the control collagen gels, suggesting that the combination of OC and OPN has a significant impact in proliferation of BM MSC. This effect was not only observed when cells were cultured using growth medium DMEM + 10% FBS, but also when cells were cultured under osteogenic differentiation conditions for 15 days. Figure 2a shows fluorescence microscopy images of DAPI and Phalloidin of BM MSC cultured after 15 days under DMEM + 10% FBS, suggesting that OC/OPN-enhanced collagen gels presented more cells compared with the control.

The number of cells after 15 days was higher when cells were cultured with DMEM + 10% FBS compared with osteogenic medium (4.54 ± 0.36 × 10^4^ cells *vs* 3.10 ± 0.11 × 10^4^ cells in OC/OPN#1 condition). The proliferation rate of BM MSC cultured onto OC/OPN-enhanced collagen gels was higher compared to control collagen gels when both media were used, demonstrating a consistent effect of the integration of OC and OPN proteins into type I collagen matrices. Indeed, after 15 days in culture under DMEM + 10% FBS, the cell number on OC/OPN#1-enhanced collagen gels was 97% higher than the number of cells on control collagen gels (4.54 ± 0.36 × 10^4^ cells *vs* 2.30 ± 0.08 × 10^4^ cells). When osteogenic differentiation medium was used, after 15 days, BM MSC presented a cell number increase of 57% when cells were cultured on OC/OPN#1-enhanced collagen gels compared to control collagen gels (3.10 ± 0.11 × 10^4^ cells *vs* 1.97 ± 0.14 × 10^4^ cells).

Interestingly, when collagen gels were supplemented with only one of the proteins (OC-enhanced collagen gel or OPN-enhanced collagen gel), more cells were observed compared to control collagen gels, after 15 days of culture, although this increase in cell proliferation was not considered statistically significant, suggesting that OC and OPN have a synergistic effect on cell proliferation.

### Effects of OC/OPN-enhanced collagen gels on osteogenic differentiation

Experiments, conducted in triplicate, showed that the expression levels of key osteogenic genes (Col I, Runx2, OPN and OC) at day 21 were significantly higher in the OC/OPN-enhanced collagen gels than in the control collagen gels, indicating that the incorporation of OC and OPN onto the collagen gels enhanced the osteogenic differentiation of BM MSC (Fig. 2d and Supplementary Fig. 1). More importantly, OC/OPN-enhanced collagen matrices promoted more osteogenic activity by sustaining the higher expression of osteogenic genes during the 21 days of culture and accelerating the osteogenic differentiation of BM MSC. In particular, when cells were cultured on OC/OPN-enhanced collagen gels, the highest level of ALP gene expression occurred 7 days earlier compared with cells cultured on control collagen matrices, indicating that osteogenic differentiation was accelerated when OC and OPN were incorporated into type I collagen gels.

OC/OPN-enhanced collagen gels also increased the expression levels of Runx2 gene after 7 days of osteogenic differentiation, reaching its maximum relative expression at day 15 and sustaining the higher expression until day 21 of culture. Control collagen gels also increased the expression of Runx2 gene on BM MSC after 7 days of osteogenic differentiation, however, the level of expression was only 30% compared with the maximum expression obtained with OC/OPN-enhanced collagen gels. Furthermore, the level of expression remained low at 20–30% compared with the maximum relative level of expression in OC/OPN-enhanced collagen gels. In contrast to collagen gel, the levels of OPN and OC genes increased throughout osteogenic differentiation, reaching higher expression levels when OC/OPN-enhanced collagen gels were used as a platform. Interestingly, after 21 days of osteogenic differentiation, OPN-enhanced collagen gels demonstrated also significantly higher expression levels of OPN and Col I gene compared with the control and even with the OC-enhanced collagen gels. On the other hand, OC-enhanced collagen gels presented significantly higher expression levels of Runx2 and OC osteogenic genes compared with the control and OPN-enhanced collagen gels. However, only OC/OPN-enhanced collagen gels demonstrated significant expression levels that were consistent for osteogenic genes including Col I, Runx2, OC and OPN (Supplementary Fig. 1) compared with the control collagen gels. In addition, OC/OPN-enhanced collagen gels presented a statistically significant higher expression of Col I, OPN and OC genes compared with the OC-enhanced collagen gels and also a statistically significant higher expression of Runx2 and OC osteogenic genes when compared with the OPN-enhanced collagen gels (Supplementary Fig. 1).

### Effects of OC/OPN-enhanced collagen gels on angiogenesis

**Figure 3:**
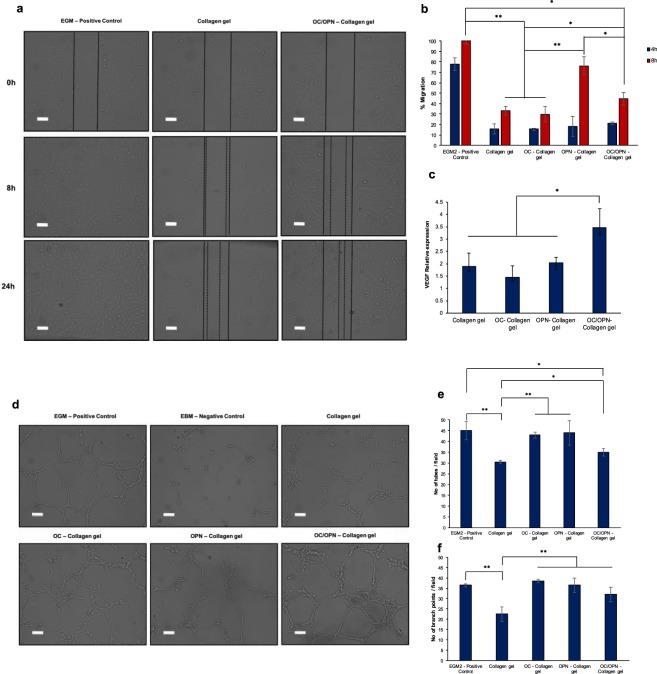
Effects of OC/OPN-enhanced collagen gels on BM MSC *in vitro* angiogenic properties assessed by multiple assays. (**a**) Cell migration assay. Scratch at t = 0 h, t = 8 h and t = 24 h when HUVEC were treated with EGM-2 (positive control), conditioned medium from BM MSC cultured on control collagen gels and conditioned medium from BM MSC cultured on OC/OPN-enhanced collagen gels. Borders of the scratch at t = 0 are indicated with solid lines, borders after migration at t = 8 and t = 24 hours with dashed lines. (**b**) Percentage of migration distance quantification of HUVEC treated with conditioned medium from BM MSC cultured on control collagen gels, OC-collagen gels, OPN-collagen gels and OC/OPN-collagen gels. (**c**) VEGF relative expression of BM MSC cultured on OC/OPN–enhanced collagen gels, OC-enhanced collagen gels, OPN-enhanced collagen gels and control collagen gels after 21 days of culture under osteogenic differentiation, normalized to VEGF relative expression of undifferentiated BM MSC. (**d–f**) Endothelial cell tube formation assay: (**d**) Tube formation of HUVEC on a Matrigel substrate incubated with EGM-2 (positive control), EBM-2 (negative control) and conditioned medium from BM MSC cultured on collagen gels, OC-collagen gels, OPN-collagen gels and OC/OPN-collagen gels. (**e**,**f**) Number of tubes/field (**e**) and number of branch points/field (**f**). Scale bars, 100 µm. Data are expressed as mean ± s.e.m.; **P < 0.01; *P < 0.05.

To evaluate whether OC/OPN-enhanced collagen gels could positively enhance the release of soluble factors that stimulate chemotaxis and angiogenesis, an *in vitro* cell migration assay using scratch wound healing and an endothelial tube formation assay were performed. Figure 3a shows that cell migration was observed after 8 hours in all groups. Under culture conditions employed in this study, human umbilical vein endothelial cells (HUVEC) treated with conditioned medium from BM MSC cultured on OC/OPN-enhanced collagen gels migrated faster than HUVEC treated with conditioned medium from BM MSC cultured on control collagen gels, suggesting that OC/OPN-enhanced collagen gels enhance migration, reaching a remarkable increase of 45% in cell migration distance after 8 hours.

Conditioned medium from OPN-enhanced collagen gels also presented a significant increase of HUVEC migration when compared with the migration distance achieved by HUVEC treated with conditioned medium from control collagen gels. After 24 hours, the initial scratch was almost completely closed when HUVEC were treated with conditioned medium from OC/OPN-enhanced collagen gels, reaching almost the same migration rate of HUVEC treated with endothelial growth medium (EGM-2), the positive control.

The endothelial cell tube formation assay showed a similar pattern to the one observed with the cell migration assay. Figure 3d shows that the addition of conditioned medium from BM MSC cultured on OC/OPN-enhanced collagen gels increased the tube formation of endothelial cells compared to conditioned medium from BM MSC cultured on control collagen gels. Quantitative analysis revealed that the number of total capillary tubes and branch points formed were significantly increased by the conditioned medium from BM MSC cultured on OC/OPN-enhanced collagen gels, indicating the favored angiogenic potential of these new biomimetic matrices (Fig. 3e,f). The results also showed that OC-collagen gels and OPN-collagen gels enhanced tube formation of endothelial cells, by the significant increase in the total number of tubes and branches points formed. Although all the three groups (OC/OPN, OC and OPN) demonstrated enhanced angiogenesis compared with the control, a more robust network of capillary-like structure was observed when conditioned medium from cells cultured on OC/OPN-enhanced collagen gels was used (Fig. 3d). We observed that the length of the tubes was slightly higher, compared with OPN and OC groups, leading to a better interconnected structure, therefore, the quantification of number of tubes and branches per field was lower with the OC/OPN group. Furthermore, the relative level of VEGF gene expression was significantly increased when BM MSC were cultured on OC/OPN-enhanced collagen gels (Fig. 3c).

### Effects of OC/OPN-enhanced collagen gels on mineralization and mineral quality

Here we sought to determine whether enhanced and accelerated osteogenic differentiation of MSC on OC/OPN-enhanced collagen gel results in deposition of mineral similar to the one produced by bone tissue, *in vivo* – a key aspect of high quality functional bone tissue and a gold standard of clinical practice (determined using periodical X-rays).

**Figure 4:**
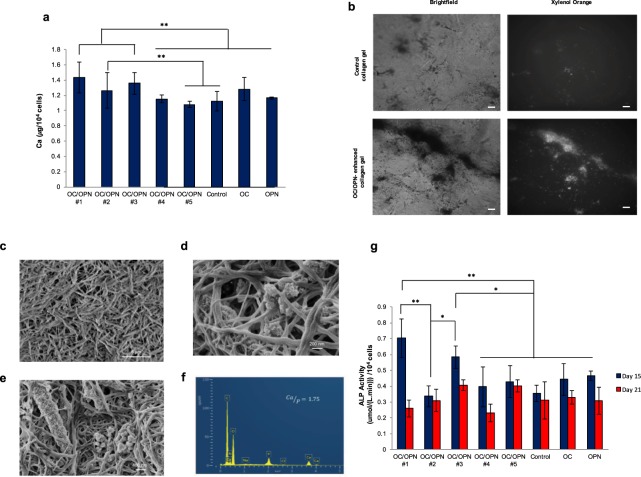
Effects of OC/OPN-enhanced collagen gels on BM MSC mineralization and ALP activity. (**a**) Calcium content quantification of BM MSC on OC/OPN-enhanced collagen gels cultured for 21 days compared to control collagen gels. (**b**) Mineralized nodules of MSC after 21 days of culturing in osteogenic differentiation medium on OC/OPN-enhanced collagen gels and control collagen gels without OC/OPN incorporation. The nodules were visualized by Xylenol orange staining. Scale bars, 100 µm. (**c–e**) SEM images of BM MSC cultured on OC/OPN-enhanced collagen gels. **(c)** OC/OPN-enhanced collagen gels before cell culture; **(d,e)** BM MSC mineralization of OC/OPN-enhanced collagen gels after 21 days of osteogenic differentiation. (**f**) EDS spectrum of mineral deposition of BM MSC cultured on OC/OPN-enhanced collagen gels. (**g**) Alkaline phosphatase (ALP) activity of BM MSC cultured on OC/OPN-enhanced collagen gels for 15 days and 21 days of differentiation. Data are expressed as mean ± s.e.m.; **P < 0.01; *P < 0.05.

We found that after 21 days of osteogenic differentiation, BM MSC demonstrated higher calcium deposition when cells were seeded onto OC/OPN-enhanced collagen gels (Fig. 4a) compared to control, OC-collagen gels and OPN-collagen gels. Moreover, statistically significance was observed when cells were cultured on OC/OPN#1, OC/OPN#2 and OC/OPN#3-enhanced collagen gels. Figure 4a shows that when BM MSC were cultured and differentiated onto OC/OPN supplemented with 3 μg/ml OC and 0.6 μg/ml OPN (OC/OPN #1), a significant increase of calcium deposition of 48% was observed compared with the control collagen gels (1.42 ± 0.11 μg/10^4^ cells *vs* 0.96 ± 0.04 μg/10^4^ cells).

Mineralized nodules of human MSC were visualized after 21 days of culturing in osteogenic differentiation medium. The nodules were visualized by Xylenol orange staining in OC/OPN-enhanced collagen gels and in control collagen gels without OC and OPN incorporation (Fig. 4b). Scanning electron microscopy (SEM) micrograph of mineral deposition of BM MSC cultured on OC/OPN-enhanced collagen gels is shown in Fig. 4c–e. After 21 days, OC/OPN-enhanced collagen gels presented pores filled with globular mineralized nodules, indicating osteoblastic differentiation.

Next, we conducted spectroscopic analysis using SEM coupled with energy dispersive spectroscopy (EDS) and found that the mineral deposition of MSC cultured on OC/OPN-enhanced collagen gels consisted of calcium and phosphate. The Ca/P ratio of minerals formed within the OC/OPN-enhanced collagen gels was ~1.75, which is comparable to apatite in bone matrix (i.e., Ca/P ~1.66)^29^, in contrast with the control collagen gels that demonstrated a Ca/P ratio of 1.35. (Fig. 4f).

ALP activity is another key osteoblast differentiation marker and mineralization. ALP activity quantitative analysis showed that OC/OPN-enhanced collagen gels (OC/OPN#1, OC/OPN#3) presented significantly higher ALP activity compared with the control collagen gels, OC-collagen gels and OPN-collagen gels at day 15 (Fig. 4g). Moreover, in OC/OPN#1-enhanced collagen gel, ALP activity was two times higher than the control at day 15 of osteogenic differentiation (0.70 ± 0.12 (μmol/(L.min))/10^4^ cells *vs* 0.35 ± 0.07(μmol/(L.min))/10^4^cells).

### Effects of OC/OPN-enhanced mineralized collagen scaffolds on local inflammatory response in a critical sized-defect rabbit long-bone model

Here we sought to determine the local inflammatory response of the OC/OPN-enhanced mineralized collagen scaffolds. Thus, we evaluated if the presence of OC/OPN-collagen scaffolds *in vivo* would promote any infection or foreign body reaction while new bone was being formed.

To this end a critical sized-defect was induced in a rabbit model followed by femoral/tibial implantation of the scaffolds. In bone, a critical sized-defect does not heal without intervention over the natural lifetime.

**Figure 5:**
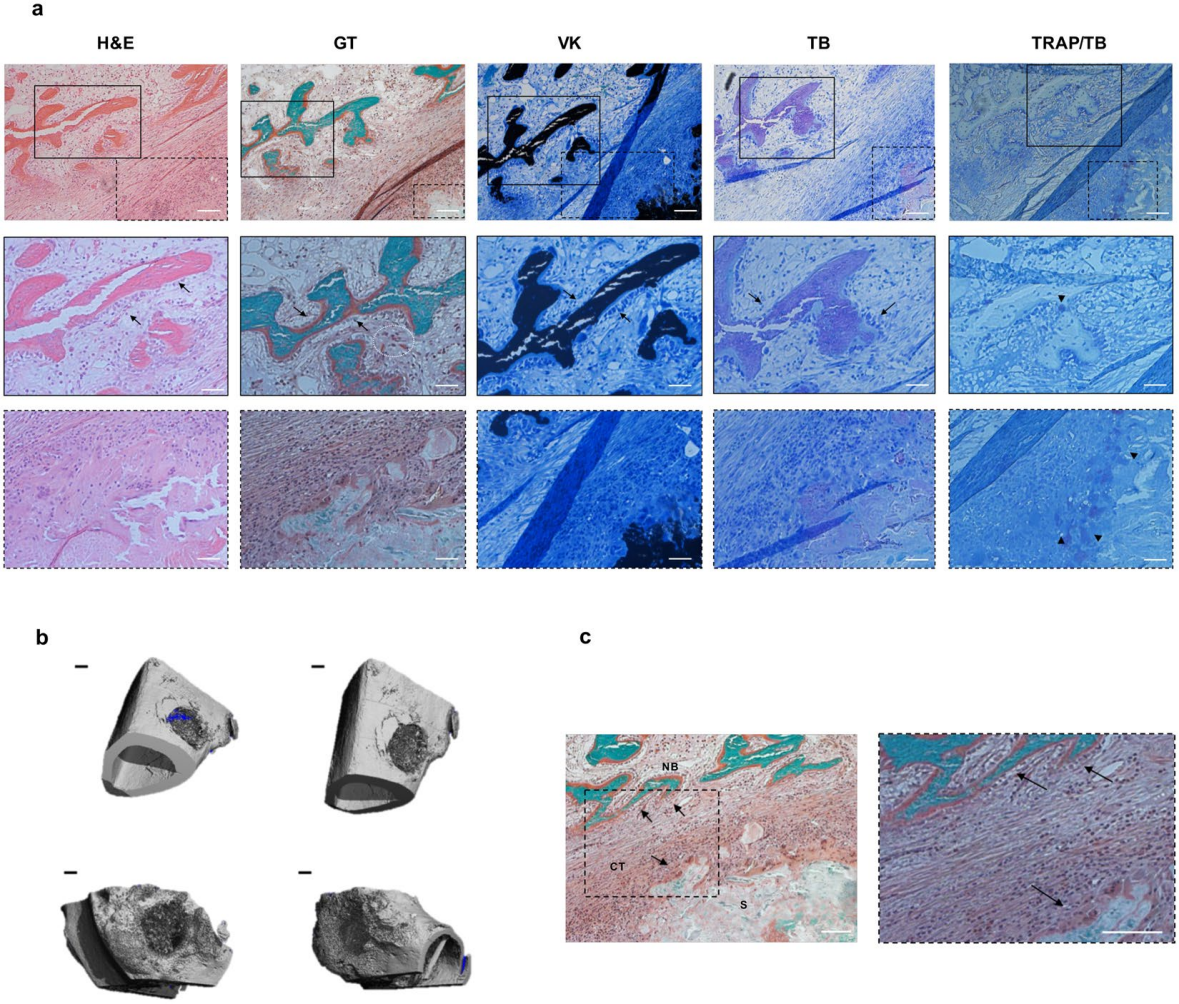
Bone regeneration in a critical sized-defect rabbit long-bone model using OC/OPN-enhanced mineralized collagen scaffolds. (**a**) Representative histological images of the rabbit critical sized-defect at 6 weeks postimplantation. Hematoxylin and Eosin (H&E), Goldner’s Trichrome (GT), Von Kossa (VK), Toluidine Blue (TB) and Tartrate-resistant acid phosphatase (TRAP) stainings. Panels on the first row are at 100x magnification. Black squares represent the area of new bone formed. Dashed squares represent the area of the implanted scaffold. Scale bars, 100 µm. Panels on the second row are at 200x magnification and represent the area of new bone formation (black squares). Scale bars, 50 µm. Panels on the third row are at 200x magnification and represent the area nearby the implanted scaffold (dashed squares). Black arrows outline the osteoblasts covering the new bone formed. Black arrowheads represent the osteoclasts by TRAP staining, indicating a possible remodeling of bone. White circle represents new vessels formed. Scale bars, 50 µm (**b**) Representative microcomputed tomography images of bone regeneration in the rabbit model at 6 weeks postimplantation. Top row represents a tibial defect, bottom row represents a femoral defect. Scale bars, 1 mm. (**c**) Goldner’s trichrome staining – detailed investigation. Detailed description of new bone formation within the OC/OPN-enhanced mineralized collagen scaffolds. NB – new bone; CT – connective tissue; S – scaffold. Scale bars, 100 µm.

Bone regeneration of a critical-sized defect was observed when OC/OPN-enhanced mineralized collagen scaffolds were implanted into a femoral/tibial defect after 6 weeks postimplantation (Fig. 5b). The surgical incision site was observed for wound healing and signs of infection daily for at least ten days following surgery. No signs of inflammation, discharge or wound dehiscence and abscess were observed. No specimen revealed any evidence of infection or foreign body reaction, and all wounds showed a good healing response.

Histological images showed new bone formation surrounding the implant area (Fig. 5a). Connective tissue was found between the new bone formed area and the implant. Osteoblasts were observed between the new mineralized bone and evidence of angiogenesis, in the form the new vessel formation, was seen. Green and purple areas observed using Goldner’s Trichrome (GT) and Toluidine Blue (TB) stains, respectively, confirmed that new bone was formed after 6 weeks post-implantation. Osteoclasts were found in the implanted scaffold via Tartrate-resistant acid phosphatase (TRAP) staining, indicating a possible initial stage of bone remodeling. However, further studies are required to confirm that bone remodeling is occurring. In addition, further studies are needed to evaluate the mechanism of action, determining if the new bone formation observed herein is due to the presence of OC/OPN or due to the collagen scaffold.

### Effects of OC/OPN-enhanced collagen gels on human MSC from different tissue sources

In order to evaluate the synergistic effect of OC/OPN with MSC from multiple sources beyond bone marrow derived MSC, we investigated responses of MSC isolated from different human tissue sources on OC/OPN-enhanced collagen gels.

**Figure 6:**
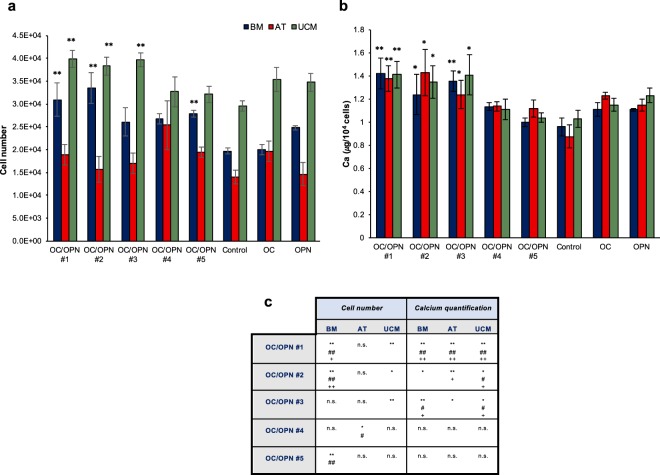
Proliferation and mineralization of human MSC from different tissue sources cultured on OC/OPN-enhanced collagen gels. (**a**) Proliferation of MSC from BM, AT and UCM on OC/OPN-collagen gels after 15 days of culture. **P < 0.01; *P < 0.05 relative to the control group for each cell source. (**b**) Calcium quantification of MSC from BM, AT and UCM cultured on OC/OPN-enhanced collagen gels after 21 days of osteogenic differentiation. Data are expressed as mean ± s.e.m, **P < 0.01; *P < 0.05 relative to the control group for each cell source. (**c**) Summary of statistically significant differences between each different composition of OC/OPN and control collagen gels, OC-collagen gels and OPN-collagen gels for each cell source (BM, AT, UCM), regarding cell number and calcium quantification after 15 days of culture. **P < 0.01, *P < 0.05 relative to control collagen gel; ++P < 0.01, +P < 0.05 relative to OC-collagen gel; ^##^P < 0.01, ^#^P < 0.05 relative to OPN- collagen gel; n.s. – not significant.

MSC from umbilical cord matrix (UCM) and adipose tissue (AT) were seeded onto the biomimetic matrices. Proliferation assay showed more cells on most of the OC/OPN-enhanced collagen gels compared with the control collagen gels for all the different tissue sources (UCM, BM and AT) (Fig. 6a). Compared to the BM MSC, AT MSC reached lower number of cells after 15 days of culture. Moreover, when OC and OPN were incorporated individually into the gel, a slight increase of number of AT MSC was seen compared with the control collagen gels. In our work, UCM MSC reached higher number of cells than BM MSC in all the biomimetic collagen gels. We observed that UCM MSC cultured on OC-collagen gels and OPN-collagen gels presented more number of cells after 15 days compared with some OC/OPN compositions, such as OC/OPN #4 and OC/OPN #5, however, these values are not statistically significant compared with the control and with the other OC/OPN compositions. Calcium quantification did not demonstrate any significant difference between cells from all the different tissue sources, however, consistent with results presented above, cells cultured on OC/OPN-enhanced collagen gels presented higher amount of calcium levels (Fig. 6b). The synergistic effect of the incorporation of OC/OPN was observed in terms of proliferation and mineralization for all the different tissue sources, showing that the effects of OC/OPN are independent of cell source.

## Discussion

The biological process of bone healing is complex and is influenced by multiple factors such as the patient’s health and nutritional conditions, and the degree and stability of the fracture experienced^4^. Overall, 5–10% of fractures demonstrate delayed or impaired healing, affecting the patient’s quality of life and leading to socioeconomic consequences and repeated surgeries^4^. Non-union fractures represent the most dramatic case in which bone healing fails in the absence of treatment. In this case, autografts or allografts can be used to treat non-union fractures, however bone grafts must consider the characteristics, localization and healing potential of the non-unions fractures, ensuring graft stability, osteogenesis and osteoinduction^1,4^. Depending on the type of non-union fractures, bone substitutes from different materials can be used to fix and repair non-union fractures. Moreover, some molecular signals have been incorporated into these materials, such as bone morphogenetic proteins (BMP) that play the leading role in the field of bone tissue engineering. However, BMP have some drawbacks, such as the high costs of production and the high doses required, raising questions about their cost effectiveness^1^. With increasing clinical use of BMP, side effects have also been reported, including postoperative inflammation, ectopic bone formation, osteoclast-mediated bone resorption and inappropriate adipogenesis^30^.

Therefore, there is an urgent need for development of new techniques that accelerate fracture healing process, enhance bone regeneration and remodeling and result in new bone tissue that is similar to human bone in structure and function. Such changes will ultimately improve patient’s quality of life and decrease the high health-care costs associated with delayed bone healing, repeated surgeries and longer rehabilitation. Biomimetic construction of engineered bone tissue using selected proteins represents a promising alternative to the use of poor quality bone grafts and BMP in order to address the increasing worldwide demand of fracture repair and bone regeneration in an aging and osteoporotic population with limited bone regeneration potential.

The present study identified a new biomimetic strategy to accelerate osteogenic differentiation and sustained bone formation response from MSC obtained from multiple tissue sources. In particular, it demonstrates, for the first time, the synergistic effect of different concentrations of bone matrix non-collagenous proteins, OC and OPN, incorporated onto type I collagen gels, showing enhancement of proliferation and acceleration of osteogenic differentiation of human MSC and angiogenesis, eventually resulting in increased mineralization and bone regeneration *in vitro*.

To mimic the *in vivo* 3D ECM of connective tissue, a 3D type I collagen matrix is often used as scaffold, since type I collagen is a predominant ECM molecule^31^. However, different tissues have a different composition of the ECM, and the composition provide specific information and clues to cells. Consequently, ECM composition of native tissue, in particular the role of key matrix proteins, should be carefully considered for the development of novel biomaterials based on collagen scaffolds^31^. Regarding bone ECM, we incorporated OC and OPN as non-collagenous extracellular bone matrix components in scaffold design as an attempt to achieve a better osteogenic potential of the gels while selectively mimicking the bone environment. Few studies have used components of the organic bone matrix other than collagen to create bone substitutes^32–35^. Here we used OC and OPN for the first time as together they provide structural integrity to bone matrix and both are lost with tissue aging leading to altered mineralization and bone formation^7,13,23,24^. Furthermore, autografts as well as allografts, typically obtained from older donors or patients that were subjected to total hip arthroplasty procedures^25^, are likely to contain bone tissue that has reduced concentrations of OC and OPN^7^ and, therefore, is structurally compromised^23^ and not fully functional to promote mineralization.

During fracture healing, the new bone formation includes three coordinated biological events: osteogenic cell recruitment and proliferation; osteogenic cell differentiation and mineralization; and vascularization of the repair site. Disruption of any one of these can lead to delayed or impaired healing^6^.

As confirmed here, biomimetic OC/OPN-enhanced collagen matrices enhanced cell proliferation, promoted early osteogenic differentiation and angiogenesis, and produced a sustained bone formation response resulting in mineralized tissue similar to bone.

OPN is commonly found surrounding mineralized tissues^36^ and has been considered to play an important role in cell attachment and in the recruitment of osteoblasts during the early stage of bone formation^37,38^. OPN can bind to α_ν_β_3_ integrins through their RGD domain^11^. Additionally, OPN can also present an RGD-independent mechanism, in which OPN may engage CD44^12^, a cell surface adhesion molecule, involved in cell-cell and cell-matrix interactions. Shin and co-workers demonstrated that OPN enhanced MSC adhesion and proliferation on biomimetic hydrogels modified with an OPN-derived peptide^39,40^. On the other hand, OC has not been reported to induce MSC proliferation, however recent studies have shown that exogenous OC is sufficient to induce myoblast^41^ and β-cell^42^ proliferation. In contrast to above, our results show that both OPN and OC are required for increased MSC proliferation on collagen gels, since OPN-enhanced collagen gels and OC-enhanced collagen gels did not demonstrate a statistically significant increase on cell proliferation. Therefore, the synergistic effect of these two proteins is required.

On the other hand, OC has affinity for calcium through the gama-carboxyglutamic acids^43^ and by binding hydroxyapatite, it can accelerate its nucleation, playing an active role in the early stage of bone healing^32^. Nucleation and growth of hydroxyapatite crystals within the collagenous matrix are two fundamental steps for bone mineralization. Therefore, OC might favor mineralization and osteogenic differentiation of MSC on OC/OPN-enhanced collagen gels. Consistent with this notion, we found that OC and OPN enhanced the osteogenic differentiation of human BM MSC cultured on the OC/OPN-enhanced collagen gels. During the immediate post-proliferative period, the extracellular matrix undergoes a series of modifications in composition and organization that makes it competent for mineralization. Therefore, after 21 days of osteogenic differentiation, OC/OPN-enhanced collagen gels presented significantly higher calcium deposition and expression of osteogenic genes (Col I, Runx2, OPN and OC) compared with the control collagen gels without OC and OPN incorporation (Figs 2 and 4 and Supplementary Fig. 1). Interestingly, OC-enhanced collagen gels also upregulated the expression of Runx2 and OC gene levels, demonstrating also better osteogenic differentiation (Supplementary Fig. 1), suggesting that osteocalcin is favoring this phenomenon.

During this post-proliferative phase, the cells also express alkaline phosphatase that reaches its peak of expression and declines as the cultures progress into the mineralization stage^44^. Interestingly, when BM MSC were cultured on OC/OPN-enhanced collagen gels, the ALP gene expression was highest at day 7 of osteogenic differentiation and then progressively declined at 15 and 21 days of osteogenic differentiation. On the other hand, when BM MSC were cultured on control collagen gels, the overall ALP gene expression was lower than OC/OPN-enhanced collagen gels reaching the highest value at day 15 of osteogenic differentiation (Fig. 2b) followed by a progressive decline. This result confirmed that BM MSC cultured on OC/OPN-enhanced collagen gels presented not only a higher extent of ALP expression but also the earlier temporal ALP gene expression after 7 days of osteogenic differentiation, indicating an acceleration in the osteogenic differentiation based on the early ALP gene expression.

OC/OPN-enhanced collagen gels also enhanced the expression levels of Runx2 gene after only 7 days of osteogenic differentiation, reaching its maximum relative expression at day 15 and sustaining the high level thereafter.

Regarding the mineralization process, several genes were induced to maximal levels, paralleling to accumulation of mineral in bone regeneration. OPN and OC are bone proteins known to be associated with the mineralized matrix *in vivo* and achieve their peak levels of expression during mineralization of the extracellular matrix^44^. As shown in our results, after 21 days of osteogenic differentiation, OC/OPN-enhanced collagen gels presented higher levels of OPN and OC gene expression, indicating the formation of a more mature extracellular matrix compared with control collagen gels without OC and OPN incorporation.

Notably, OC-enhanced collagen gels and OPN-enhanced collagen gels were able to upregulate osteogenic genes. OPN-enhanced collagen gels demonstrated upregulation of Col I and OPN genes, however Runx2 and OC did not show any statistically significant increase. On the other hand, OC-enhanced collagen gels demonstrated enhancement of Col I, Runx2 and OC genes but did not show improvement of OPN gene levels. Moreover, OC and OPN genes were only both upregulated when MSC were cultured on OC/OPN-enhanced collagen gels (Supplementary Fig. 1), indicating that a possible positive feedback loop might be involved in this phenomenon.

These results of higher and sustained osteogenic gene expression of OC/OPN-enhanced matrices were associated with more accumulation of mineral, and the mineralized nodules contained a Ca/P ratio comparable to native bone matrix. Close correspondence of Ca/P ratio between the regenerated and natural bone tissue provides supporting evidence on the quality of new formed bone. For example, in osteogenesis imperfecta patient biopsies, a condition derived from mutation in type I collagen, the Ca/P ratio was shown to be lower than normal bone, leading to imperfect bone formation, demonstrating a compromised bone quality and bone fragility^45^. To our knowledge, very few studies have reported similar information on newly regenerated bone.

Furthermore, in contrast to OC or OPN-enhanced matrices, optimal levels of mineralization were obtained when MSC were seeded on collagen gels incorporated with both OC and OPN, suggesting that these proteins act in a synergistic manner. Therefore, we hypothesize that the enhancement of osteogenic differentiation of BM MSC on OC/OPN-enhanced collagen gels observed herein might be due to the presence of both OC/OPN in the matrix. In fact, the mechanisms responsible for bone formation and remodeling likely involve the association of bone matrix proteins into specific complexes that helps the organization of the matrix^46^.

Previous studies have shown that these two proteins have the ability to interact^46^. In fact, Ritter and colleagues have investigated the association of OPN with OC using three different ligand binding techniques, indicating that OPN specifically associates with OC^46^, forming stable complexes, however their metabolic role was not investigated. OC has been shown to bind strongly to the bone mineral hydroxyapatite and it complexes with and links to collagen through OPN^46^. In this study, results shown by ELISA measurements demonstrated that OC/OPN-enhanced collagen matrices released a negligible amount of OC and OPN, indirectly proving the association between these proteins and collagen (Supplementary Fig. 3).

Both OC and OPN have sequence motifs that could allow each protein to interact with other molecules. Through its γ-carboxyglutamate residues, OC forms a calcium binding pocket^47^. It has been suggested that via this pocket, OC can bind to other calcium binding proteins. Moreover, the COOH terminus of OC can also participate in protein binding, since it adopts a *β*-sheet conformation that is exposed even when the molecule is bound to hydroxyapatite. OPN also comprises motifs that would allow it to interact with other proteins. It has an integrin binding RGD sequence that functions in cell attachment^48^. Therefore, this RGD sequence present in OPN might be the trigger to the enhancement of cell proliferation. Also, OPN is a substrate for transglutaminase activity^49^, a reaction that can produce complexes between proteins *in vivo*. In this study, we did not evaluate, directly, the interaction of both proteins, however we were able to observe better biological responses from BM MSC when these cells were seeded on OC/OPN-collagen gels, compared with only OC-collagen gels and OPN-collagen gels. Our data suggests that the presence of both proteins in the matrix enhanced cellular responses, such as proliferation and osteogenesis, and supported the evidences reported by Ritter and colleagues regarding the metabolic effects of the presence of OC/OPN.

Previous work from our group found that fracture in bone initiates as dilatational bands that form as a result of OC-OPN interaction. In the absence of either protein, the complex is disrupted, resulting in a loss of the structural integrity of bone matrix^23^. However, further studies need to be done to understand better how these two proteins interact within the collagen matrix.

After implantation of biomimetic scaffolds in a defect, cell migration is a critical step for bone regeneration. In the present study, experiments demonstrated that conditioned medium from BM MSC seeded on biomimetic OC/OPN-enhanced collagen gels stimulated a faster migration of HUVEC. Notably, conditioned medium from OPN-collagen gels also demonstrated a faster migration of HUVEC, even when compared with the results obtained with conditioned medium from cells cultured on OC/OPN-enhanced gels. In fact, OPN alone has been reported to improve cell migration and wound healing response^50^, a phenomenon that is linked with the migration potential of cells. However, it is not yet clear which soluble factors and mechanisms are responsible for stimulating the migration of cells. Indeed, MSC have been described as inducers of wound healing and angiogenesis and they secrete paracrine factors that indirectly initiate repair following injury^51^. Bone is a highly-vascularized tissue and for bone regeneration an adequate blood flow is required to provide sufficient supply of nutrients and oxygen to the cells^52,53^. In response to angiogenic signals found in conditioned media obtained from BM MSC cultures, endothelial cells may form capillary like structures^54^. In our study, conditioned medium from BM MSC seeded onto OC/OPN-enhanced collagen gels increased the tube formation of endothelial cells, when compared with the control, by increasing the number of total capillary tubes and branch points formed. We believe that two different mechanisms may be responsible for the improvement of angiogenic properties demonstrated by OC/OPN. Indirectly, the increase in cell number present in the OC/OPN-enhanced collagen gels might be responsible for a higher concentration of angiogenic soluble factors released from cells. On the other hand, the significant increase of mRNA levels of VEGF by BM MSC cultured on OC/OPN-enhanced collagen gels demonstrated that the enhancement of expression levels of VEGF might be, directly, improving the angiogenic properties. These results confirm that the effect of OC/OPN on angiogenesis occurs with stimulating the release of angiogenic factors and enhancing the VEGF gene expression. OC-enhanced collagen gels and OPN-enhanced collagen gels did not promote an increase in the expression of VEGF mRNA levels by BM MSC, demonstrating the synergistic effect of both proteins in angiogenesis. However, further studies need to be done to quantify and identify the angiogenic factors secreted by these cells that were exposed with OC/OPN.

Consistent with our above proposal, previous studies showed that the presence of OPN induces HUVEC proliferation, survival and migration resulting in tube formation and VEGF expression^55–59^. Regarding the effect of OC on angiogenesis, Cantatore and colleagues showed that OC alone, exogenously applied to chick embryo chorioallantoic membrane, stimulates angiogenesis^60^. Although our results demonstrated that OC-collagen gels and OPN-collagen gels also enhanced tube formation and migration of endothelial cells, we demonstrated that by applying the synergistic strategy of both proteins onto the collagen gels, both osteogenesis and angiogenesis processes were enhanced. Thus, the incorporation of OC or OPN enhanced individual effects on cells, however an overall significant enhancement on different cellular processes, such as cell proliferation, osteogenic differentiation and angiogenesis, was found with OC/OPN-enhanced matrices. Of note, our study evaluates for the first time the effect of both non-collagenous proteins, used in combination in a biomimetic collagen gel, on the angiogenic capacity of human MSC.

We also evaluated and compared the influence of OC/OPN-enhanced collagen gels on MSC derived from different sources: UCM, BM and AT. MSC derived from all three tissues showed enhanced proliferation and mineralization results when OC/OPN were incorporated into the biomimetic collagen gels. UCM MSC have been shown to exhibit superior proliferative capacity^61^. Our work confirmed that these cells reached higher number of cells than BM MSC in all the biomimetic collagen gels. Although some differences in proliferation were observed between MSC from different tissues, here we demonstrate that the effect of OC/OPN on the *ex-vivo* proliferation and osteogenic potential of MSC is not tissue source dependent, indicating a great potential to translate these findings to a clinical context.

*In vivo* data using a critical sized-defect rabbit long-bone model revealed that OC/OPN-enhanced mineralized collagen scaffolds did not promote any adverse reaction, while new bone was being formed after 6 weeks of implantation in a femoral/tibial defect. Moreover, osteoblasts and blood vessels were found surrounding the new bone formed (Fig. 5a).

OC/OPN-enhanced collagen matrices, developed here, can be applied in other systems in which the production of OC and OPN might be compromised, due to medical conditions or to the age of the patient. In fact, variation of non-collagenous bone protein concentrations in diseased human bones have already been reported^22^. Findings to date are that osteocalcin and osteopontin levels are reduced in osteoporotic bone^62^ and in older bone tissue^7^. Moreover, it was reported that older people may have a 10-fold increased 10-year fracture risk compared with younger people with the same bone mineral density^63^. Therefore, with aging there is an increased susceptibility to fractures due to the increase in skeletal fragility. We believe that, since these proteins are important for fracture resistance^64^, OC/OPN-enhanced collagen matrices might help to sustain bone formation when patients are not or less able to produce naturally these proteins, producing high quality functional bone to improve bone regeneration.

Taken together, our results demonstrate, for the first time, the significant synergistic impact of OC and OPN on proliferation, osteogenic differentiation and angiogenic capacity of MSC engineered within a combined type I collagen matrix. Further studies need to be done to evaluate the efficacy of these matrices in bone tissue engineering and regenerative medicine as biomimetic scaffolds that accelerate bone healing.

## Methods

### Preparation of OC/OPN-enhanced type I collagen matrices

To design the biomimetic OC/OPN-enhanced collagen gels, we used type I collagen at 3 mg/ml and we based on the physiological levels that Cairns and Price reported in bone, 1 mg OC per 1 g of collagen^29^ (3 µg/ml of OC). Physiologically, the content of OPN is known to be lower than OC. To optimize our results, different concentrations of OC and OPN were tested, by varying the amount of OC and OPN incorporated into the type I collagen gels (Fig. 1c), above and below bone matrix physiological levels.

Recombinant human OPN protein was purchased from R&D systems and human OC fragment 1–49 was purchased from Sigma-Aldrich. Different concentrations of OC and OPN (Fig. 1c) were combined to 100 μl of chilled purified bovine type I collagen solution (PureCol: Advanced BioMatrix, San Diego, CA) at a final concentration of 3 mg/ml with gentle swirling. pH of mixture was adjusted to 7.2–7.6 using sterile 0.1 M NaOH (Sigma-Aldrich, St. Louis, MO). To prevent gelation, the temperature of mixture was maintained at 2–10 °C. 100 μl of OC/OPN-enhanced collagen solution was added to each well from a 96-well plate. To form gel, the plate was incubated at 37 °C for 2 h. After incubation, the OC/OPN– enhanced collagen gels were hydrated in culture medium 1 h at 37 °C, prior to cell culture.

To evaluate the amount of proteins released from the collagen matrices, phosphate buffer saline (PBS: Gibco, Grand Island, NY) was added to the OC/OPN-enhanced collagen gels (all the combinations) and to the OC and OPN-collagen gels for 24 h and 21 days. After that, the PBS in contact with the matrices was collected and the concentrations of OC and OPN were measured by enzyme-linked immunosorbent assays (ELISA: R&D Systems, Minneapolis, MN), according to the manufacturer’s instructions contained in the human OPN or OC quantikine ELISA kit (R&D Systems).

For the *in vivo* studies, bovine type I collagen fibrous sheets (Advanced Biomatrix) were mineralized in the presence of OC/OPN, to produce OC/OPN-enhanced mineralized collagen matrices. Therefore, the scaffolds (6 × 6 × 5 mm^3^) were incubated, first, in a 1 M CaCl_2_ solution in the presence of 60 µg/ml OC, then with12 µg/ml OPN at 37 °C (pH = 7.4). The scaffolds were washed twice with PBS and freeze-dried. Next, the scaffolds were incubated with 1 M Na_2_HPO_4_ at 37 °C (pH = 7.4) for 24 hours and freeze-dried. The mineralized scaffolds were sterilized using ethylene oxide (Fig. 1b).

### Cell culture

Human MSC used are part of the cell bank available at the Stem Cell Engineering Research Group (SCERG), Institute for Bioengineering and Biosciences (iBB) at Instituto Superior Técnico (IST). MSC were previously isolated/expanded according to protocols previously established at iBB-IST^61,65,66^. Originally, human tissue samples were obtained from local hospitals under collaboration agreements with iBB-IST (bone marrow: Instituto Português de Oncologia Francisco Gentil, Lisboa; adipose tissue: Clínica de Todos-os-Santos, Lisboa; umbilical cord: Hospital São Francisco Xavier, Lisboa, Centro Hospitalar Lisboa Ocidental, Lisboa). All human samples were obtained from healthy donors after written informed consent according to the Directive 2004/23/EC of the European Parliament and of the Council of 31 March 2004 on setting standards of quality and safety for the donation, procurement, testing, processing, preservation, storage and distribution of human tissues and cells (Portuguese Law 22/2007, June 29), with the approval of the Ethics Committee of the respective clinical institution. Isolated cells were kept frozen in liquid/vapour nitrogen tanks until further use.

Human MSC from different sources were thawed and plated on T-75 flasks using low-glucose Dulbecco’s Modified Eagle Medium (DMEM: Gibco) supplemented with 10% fetal bovine serum (FBS MSC qualified: Gibco) and 1% antibiotic-antimycotic (Gibco) and kept at 37 °C and 5% CO_2_ in a humidified atmosphere. Medium renewal was performed every 3–4 days. Cells between passages 3 and 6 were used. Three independent donors from each tissue source were used on all experiments (ages between 30 and 40 for BM and AT donors). The cells were tested for expression of cell surface markers indicative of MSC (i.e., CD73^+^, CD90^+^, CD105^+^, HLA-DR^−^, CD34^−^, CD45^−^), and their ability to differentiate into osteoblasts, adipocytes, and chondrocytes. HUVEC were purchased from Lonza (Basel, Switzerland) and maintained in commercial Endothelial Growth Medium-2 (EGM-2: Lonza).

Three different MSC cell donors were used for each experiment. Each experiment was performed in triplicates.

### *In vitro* osteogenesis on OC/OPN-enhanced collagen gels

Human MSC were enzymatically lifted from their plates using TrypLE™ solution (Gibco) and re-suspended at 10 000 cells/cm^2^. Then, 100 μl of the cell suspension was seeded onto the confined area of the collagen gel and the cells were allowed to attach to the collagen gel for 24 h. After 24 h, the medium was removed and the non-adherent cells were washed twice with PBS. DMEM + 10% FBS was added to the cell culture on OC/OPN-enhanced collagen gels to allow cell expansion. After 5 days, osteogenic differentiation medium (StemPro® Osteogenesis Differentiation Kit: Gibco) was added to each collagen gel to induce osteogenic differentiation. Medium was changed every 3–4 days. The time at which the osteogenic medium was added is referred to as day 0 and cell culture was maintained for more 21 days after adding the osteogenic medium. The area covered by cells was visualized using a fluorescence microscope (Olympus IX51 Inverted Microscope: Olympus America Inc., Melville, NY) at a magnification of 100x, and recorded by an attached digital camera. After 21 days of osteogenic differentiation, samples were stained with Xylenol orange (Sigma-Aldrich) for calcium phosphate mineral deposits and images were acquired.

### Cell proliferation

The effect of OC/OPN-enhanced collagen gels on MSC proliferation was evaluated using AlamarBlue® cell viability reagent (Molecular Probes, Eugene, OR) (n = 3). AlamarBlue® cell viability reagent was added to the cells and incubated at 37 °C in 5% CO_2_ chamber for 2.5 h. Fluorescence was quantified (560 nm – 590 nm) and compared to a calibration curve to access the metabolic activity of viable cells under each condition. Cell proliferation was measured in triplicates in all groups. The capacity of OC/OPN- enhanced collagen gels to promote BM MSC proliferation was assessed after 5, 10 and 15 days of culture using DMEM + 10% FBS and also osteogenic differentiation medium as culture medium.

To assess cell morphology, after 24 h and 15 days of proliferation, cells were washed twice with PBS, fixed with 4% PFA for 20 min and then permeabilized with 0.1% Triton X-100 for 10 min. After permeabilization, cells were incubated with phalloidin (Invitrogen) (dilution 1:250, 2 μg/ml) for 45 min in the dark. Then, cells were washed twice with PBS and counterstained with DAPI (Invitrogen) (1.5 μg/ml) for 5 min and then washed with PBS. The fluorescent staining was imaged by fluorescence microscope (Olympus IX51 Inverted Microscope: Olympus America Inc., Melville, NY) and recorded by an attached digital camera.

### qRT-PCR analysis

Total RNA was extracted with an RNeasy Mini Kit (QIAGEN, Hilden, Germany). cDNA was synthesized from 10 ng of total RNA using iScript^TM^ Reverse Transcription Supermix (Bio-Rad, Hercules, CA). Reaction mixtures (20 μl) were incubated in a thermal cycler (Veriti 96-well thermal cycler: Applied Biosystems, Foster City, CA) for 5 min at 25 °C, 30 min at 42 °C and 5 min at 85 °C and then were maintained at 4 °C. The sequences of the specific primer sets used are given in Table 1.

**Table 1:** Sequences of primers used for qRT-PCR analysis.

Genes	Sequences
GAPDH	For: 5′ AAC AGC GAC ACC CAC TCC TC
	Rev: 5′ CAT ACC AGG AAA TGA GCT TGA CAA
Col I	For: 5′ CAT CTC CCC TTC GTT TTT GA
	Rev: 5′ CCA AAT CCG ATG TTT CTG CT
Runx2	For: 5′ AGA TGA TGA CAC TGC CAC CTC TG
	Rev: 5′ GGG ATG AAA TGC TTG GGA ACT
ALP	For: 5′ ACC ATT CCC ACG TCT TCA CAT TT
	Rev: 5′ AGA CAT TCT CTC GTT CAC CGC C
OPN	For: 5′ ATG AGA TTG GCA GTG ATT
	Rev: 5′ TTC AAT CAG AAA CCT GGA A
OC	For: 5′ TGT GAG CTC AAT CCG GCA TGT
	Rev: 5′ CCG ATA GGC CTC CTG AAG C
VEGF	For: 5′ GGA GGA GGG CAG AAT CAT CAC
	Rev: 5′ GGT CTC GAT TGG ATG GCA GT

The quantitative reverse transcription-polymerase chain reaction (qRT-PCR) was performed using SYBR® Green PCR Master Mix (Applied Biosystems) and StepOnePlus real-time PCR system (Applied Biosystems). All reactions were carried out at 95 °C for 10 min, followed by 40 cycles of 95 °C for 15 sec and 60 °C for 1 min; all were performed in triplicate. Glyceraldehyde 3-phosphate dehydrogenase was used as internal control to normalize differences in total RNA levels in each sample. A threshold cycle (Ct) was observed in the exponential phase of amplification, and quantification of relative expression levels was performed with the use of standard curves for target genes and endogenous control. Geometric means were used to calculate the ΔΔCt values and are expressed as 2^−ΔΔCt^. The mean values from triplicate analysis were compared. The value of each control sample (undifferentiated cells) was set as 1 and was used to calculate the fold difference in the target gene.

### Calcium quantification assay

For determination of total calcium content, samples (n = 3) were washed twice with PBS (Gibco) and extracted off a well of a 96-well plate in 0.5 M HCl solution (Sigma-Aldrich). Accumulated calcium was removed from the cellular component by shaking overnight at 4 °C. The consequent supernatant was utilized for calcium determination according to the manufacturer’s instructions contained in the calcium colorimetric assay kit (Sigma-Aldrich). Total calcium was calculated from calcium standard solution prepared in parallel. Absorbance at 575 nm was measured for each condition and normalized to the total number of cells, after 21 days of osteogenic differentiation.

### Alkaline phosphatase activity

Alkaline phosphatase (ALP) activity was detected using a colorimetric ALP kit (BioAssays Systems, Hayward, CA) according to the manufacturer’s protocol. Samples (n = 3) were washed with PBS (Gibco) and were incubated in the lysis buffer (0.1% Triton X-100 in PBS) by shaking for 30 min at room temperature. The lysate was added to p-nitrophenyl phosphate solution (10 mM) provided with the ALP kit. The absorbance was measured at 405 nm and normalized to the total number of cells in each sample. ALP activity assay was performed after 15 and 21 days of osteogenic differentiation.

### Cell migration assay

24-well tissue culture plates were collagen-coated by incubation in 0.2 mg/ml of collagen type I solution (Sigma) for 2 h at 37 °C before rinsing with PBS (Gibco). Each well was seeded with human umbilical vein endothelial cells at 10 000 cells/cm^2^ and maintained at 37 °C and 5% CO_2_ for 48 h to allow cell adhesion and the formation of a confluent monolayer. These confluent monolayers were then scratched with a sterile pipette tip, creating a scratch (wound) of approximately 0.25–0.3 mm in width. After creating the scratch, culture medium was then removed and replaced with conditioned medium which had been generated from BM MSC cultured for 4 days at 10 000 cells/cm^2^ using growth medium DMEM + 10% FBS on (i) control collagen gels without OC and OPN incorporation, (ii) OC-enhanced collagen gels, (iii) OPN-enhanced collagen gels and (iv) OC/OPN – enhanced collagen gels (OC/OPN#1). All scratch assays were performed in triplicate.

Migration of HUVEC was monitored by collecting images at various time intervals (0 h, 4 h, 8 h and 24 h) after the scratch was performed (Leica DM IL LED with EC3 camera system). The migration distance was quantified with the use of ImageJ (NIH) software, measuring the width of the scratch at previously defined points along its length (top, middle and bottom of the field of view). Data has been presented as extent of the cell migration, i.e. the percentage by which HUVEC migrate for each given time point compared with the original scratch width.

### *In vitro* endothelial cell tube formation assay

To evaluate the angiogenic potential of the OC/OPN-enhanced collagen gels, conditioned medium obtained from BM MSC cultures on biomimetic collagen gels was collected and used to test the tube formation assay by culturing human umbilical vein endothelial cells on a Matrigel substrate (50 μl/well) (Corning, Corning, NY). HUVEC (2 × 10^4^ cells) were cultured in a 96-well plate with 100 μl/well (4 wells per group) of conditioned medium from BM MSC cultures on (i) control collagen gels without OC and OPN incorporation, (ii) OC-enhanced collagen gels, (iii) OPN-enhanced collagen gels and (iv) OC/OPN – enhanced collagen gels (OC/OPN#1). As a negative control, HUVEC were incubated with endothelial basal medium (EBM-2: Lonza) and, as a positive control, HUVEC were incubated with endothelial growth medium (EGM-2: Lonza). After incubation for 8 h at 37 °C, three photomicrographs per well were taken under light microscopy (Leica DM IL LED with EC3 camera system). The number of branch points and tubes formed were counted with the use of ImageJ software.

### *In vivo* bone regeneration in a critical sized-defect rabbit long-bone model

New Zealand White female rabbits weighting more than 3.25 kg were used to assess the *in vivo* bone forming capacity of OC/OPN-enhanced collagen matrices and its biocompatibility. The rabbits were anesthetized with a subcutaneous injection of ketamine (35 mg/kg body weight) and acepromazine (0.75 mg/kg body weight). Bilateral surgery was performed to each rabbit (n = 3) to create a critical sized- defect in the distal femoral condyle and proximal tibia. A medial skin incision of approximately 4 cm was made in the rabbit’s hind leg. The skin was retracted laterally to allow for a lateral arthrotomy of the stifle joint. Once the femur was exposed, a lateral arthrotomy was performed and the joint opened. A transcondylar defect (approximately 6 mm diameter by 12 mm deep) was created in the femur. The point of drilling was located by finding the midpoint of the lateral condyle from the lateral fabellae to the most anterior portion of the lateral trochlea. A unicortical defect (approximately 5 mm diameter by 10 mm deep) was created in the proximal tibia at the level of the tibial tuberosity. The point of drilling was the medial side, level with the tibial tuberosity. After creating the defect, the site received a final rinse with saline to remove any residual particulate and the OC/OPN-enhanced collagen matrices were implanted in both femur and both tibia and the soft tissues and skin were closed using non-absorbable sutures. The animals were sacrificed 6 weeks after implantation. The retrieved specimens were fixed in formalin solution and prepared for micro-computed tomography and histological analysis. The specimens were embedded in methyl methacrylate and sections were cut and subjected to staining for Hematoxylin and Eosin (H&E), Goldner’s Trichrome (GT), Von Kossa (VK), Toluidine Blue (TB) and Tartrate-Resistant Acid Phosphatase (TRAP)/TB. Digital images were captured with an Olympus IX51 inverted microscope and associated with a digital camera. All methods were carried out in accordance with relevant guidelines and regulations. All animal experimental protocols were approved by Institutional Animal Care and Use Committee (IACUC) at Spring Valley Laboratories (IACUC approval number: SVL-315).

### Scanning electron microscope evaluation

Scanning electron microscope attached with an energy dispersive electron probe X-ray analyzer (SEM-EDS: Carl Zeiss ultra 1540 dual beam FIB/SEM system) was used to observe collagen gels fibrils and mineral deposition on OC/OPN-enhanced collagen gels after MSC culture. The cell cultured samples were rinsed with PBS and then fixed in 2% glutaraldehyde for 5 min, after which they were dehydrated in a graded series of ethanol and dried in a critical CO_2_ freeze dryer (Tousimis Autosamdri-815). After sputter-coating with gold, the specimens were examined at an accelerating voltage of 2.5–5 kV.

### Micro-computed tomography (µ-CT) evaluation

Tomograms of cylindrical pins were acquired and three-dimensional reconstruction was performed using an X-ray scanner (*Viva*CT 40, Scanco Medical) with a voxel resolution of ~10 µm. The parameters for the scans were 635 projections, 199 ms exposure time, 70 Kvp and 112 μA current. Images were reconstructed using the SCANCO software.

### Statistical analysis

Each experiment was conducted in triplicate. Statistical analysis of the data was performed using one-way ANOVA at the same timepoint, using GraphPad Prism version 7 software. The statistical significance of results is reported at 95% confidence intervals (P < 0.05).

## Electronic supplementary material

Supplementary Figures

## Data Availability

All data generated or analyzed during this study are included in this published article and its Supplementary Information files.
