# Supplementary material for: Biomimetic matrices for rapidly forming mineralized bone tissue based on stem cell-mediated osteogenesis
Source: Sci Rep. 2018 Sep 26;8:14388. doi: 10.1038/s41598-018-32794-4 (PMC6158243; doi:10.1038/s41598-018-32794-4)
Supplement: Supplementary file 1 — Supplementary Figures [file 41598_2018_32794_MOESM1_ESM.docx]

**Biomimetic matrices for rapidly forming mineralized bone tissue based on stem cell-mediated osteogenesis**

**Marta S. Carvalho^1,2^, Atharva A. Poundarik^1^, Joaquim M.S. Cabral^2,3^, Cláudia L. da Silva^2,3^ and Deepak Vashishth^1^**

^1^Center for Biotechnology and Interdisciplinary Studies, Department of Biomedical Engineering, Rensselaer Polytechnic Institute, Troy, NY, USA

^2^Department of Bioengineering and iBB – Institute of Bioengineering and Biosciences, Instituto Superior Técnico, Universidade de Lisboa, Lisboa, Portugal.

^3^The Discoveries Centre for Regenerative and Precision Medicine, Lisbon Campus, Instituto Superior Técnico, Universidade de Lisboa, Lisboa, Portugal

***Supplementary Figure 1.*** *Relative gene expression (collagen I [Col I], runt-related transcription factor 2 [Runx2], osteopontin [OPN], osteocalcin [OC] and alkaline phosphatase [ALP]) by BM MSC upon culture for 7, 15 and 21 days on control collagen gels, OPN - enhanced collagen gels, OC – enhanced collagen gels and OC/OPN – enhanced collagen gels. Data are expressed as mean ± s.e.m.; **P<0.01; *P<0.05*, relative to day 21 (Col I, Runx2, OPN, OC) and day 7 (ALP).

***Supplementary Figure 2.*** SDS-PAGE protein gel of collagen gel, OC- collagen gel, OPN- collagen gel and OC/OPN- collagen gel confirms the presence of OC/OPN in the matrices. White box shows bands referred to molecular weight of OPN. White dashed box shows bands referred to molecular weight of OC.

***Supplementary Figure 3.***  *In vitro* OC and OPN release from OC/OPN-enhanced collagen gels. OC and OPN levels in PBS were determined by ELISA measurements after 24 h and 21 days. Results demonstrated that the biomimetic gels did not release OC and OPN proteins, being able to maintain the proteins on the gel.
